# Patient related factors in frequent readmissions: the influence of condition, access to services and patient choice

**DOI:** 10.1186/1472-6963-10-216

**Published:** 2010-07-21

**Authors:** Sue E Kirby, Sarah M Dennis, Upali W Jayasinghe, Mark F Harris

**Affiliations:** 1 Centre for Primary Health Care & Equity, School of Public Health & Community Medicine, University of New South Wales, UNSW, Sydney 2052, NSW, Australia

## Abstract

**Background:**

People use emergency department services for a wide variety of health complaints, many of which could be handled outside hospitals. Many frequent readmissions are due to problems with chronic disease and are preventable. We postulated that patient related factors such as the type of condition, demographic factors, access to alternative services outside hospitals and patient preference for hospital or non-hospital services would influence readmissions for chronic disease. This study aimed to explore the link between frequent readmissions in chronic disease and these patient related factors.

**Methods:**

A retrospective analysis was performed on emergency department data collected from a regional hospital in NSW Australia in 2008. Frequently readmitted patients were defined as those with three or more admissions in a year. Clinical, service usage and demographic patient characteristics were examined for their influence on readmissions using multivariate analysis.

**Results:**

The emergency department received about 20,000 presentations a year involving some 16,000 patients. Most patients (80%) presented only once. In 2008 one hundred and forty four patients were readmitted three or more times in a year. About 20% of all presentations resulted in an admission. Frequently readmitted patients were more likely to be older, have an urgent Triage classification, present with an unplanned returned visit and have a diagnosis of neurosis, chronic obstructive pulmonary disease, dyspnoea or chronic heart failure. The chronic ambulatory care sensitive conditions were strongly associated with frequent readmissions. Frequent readmissions were unrelated to gender, time, day or season of presentation or country of birth.

**Conclusions:**

Multivariate analysis of routinely collected hospital data identified that the factors associated with frequent readmission include the type of condition, urgency, unplanned return visit and age. Interventions to improve patient uptake of chronic disease management services and improving the availability of alternative non-hospital services should reduce the readmission rate in chronic disease patients.

## Background

Emergency departments aim to provide treatment for more urgent and serious conditions. In Australia, the role of emergency departments has been specified as "prevention, diagnosis and management of acute and urgent aspects of illness and injury affecting patients of all age groups with a full spectrum of undifferentiated physical and behavioural disorders"[1]. However, many people who access emergency departments have complex social needs as well as a clinical condition requiring treatment [2-4]. Emergency departments across the world are reporting serious overcrowding resulting in lengthy waits which impair the quality of care and patient outcomes [5-7]. Investigation of patient profiles and the reasons behind the choice of emergency department services by frequent attenders and frequently readmitted patients is vital to guide the development of policy and design of interventions to address more appropriate patient management strategies and to prevent overcrowding [8].

Overcrowding of emergency departments has spawned a plethora of research on frequent emergency department users. Byrne et al [2] reported that frequent emergency department users were more likely to be males from low socioeconomic backgrounds with severe psychosocial problems who have a high morbidity and mortality. There have been similar findings from researchers in the UK [9-12], Canada [12,13], Sweden [14-16], USA [17-25], Italy [26], Taiwan [27,28] and Australia [29,30].

Frequently readmissions, however, have received less research attention. The Patients At Risk of Readmission (PARR) tool, used extensively in the UK for identification of patients for case management to prevent readmissions, [31,32] indicates that age, sex, ethnicity, number of previous admissions, and clinical condition are associated with readmissions. Howell *et al *[33] in a 2009 study in Australia identified age, co-morbidities, economic disadvantage, number of previous admissions as risk factors for frequent readmissions through a statistical algorithm derived from inpatient data. The Australian model was only moderately successful because of the relatively high number of false negatives.

Andersen's model of health service utilisation [34-36] provides a theoretical framework for thinking about why patients are frequently readmitted. This model postulates that health service utilisation is dependent on a range of factors such as the environment, population characteristics and health behaviours resulting in outcomes such as perceived health status, evaluated health status and consumer satisfaction. The environment includes available services and access issues. Population characteristics include demographic details, age, gender, cultural background, needs and resources. Health behaviours are influenced by personal attitudes and beliefs. Identifying the clinical, service utilisation details and demographic patient characteristics available from hospital data can identify risk factors for frequent readmissions and thereby help to develop strategies to divert these patients away from hospitals where clinically possible. The direct application of this model to emergency department utilisation summarised by Padgett *et al *[37,38], who suggests that only about 15% of emergency visits in the USA are for life threatening reasons and that poor mental health, anxiety about health combined with symptoms and injury influence people to decide to use the emergency department.

How important is access to primary care in readmissions? Hospitalisations for ambulatory care sensitive (ACS) conditions is used an outcome indicator for access to primary care [39]. It is acknowledged that treatment of ACS conditions outside hospitals, including chronic disease, reduces hospitalisations [40]. It would be expected that ACS conditions would contribute to frequent readmissions in areas with limited access to primary care services.

The problem that a small number of emergency department users account for a disproportionate use of scarce resources has been reported since the 1980s [41,42] and continues to plague the health system [43]. An Australian survey of emergency departments indicated that patients awaiting admission are a major contributing factor in emergency department overcrowding [44]. Two Australian States, NSW and Victoria, report diversion of chronic disease patients to chronic disease management services, including those provided by primary care, can reduce readmission rates [25,45].

We know little about the reasons behind the choice of emergency department services over other alternative medical services in the community. However, studies in NSW Australia have reported that patients came to the emergency department because they thought their condition warranted urgent services, with access to a doctor and tests or x-rays done in the same place but there was a mismatch between the views of clinicians and patients [46,47]. In a qualitative study performed in the USA [48], patients reported that they were unable to obtain an appointment with a primary care provider; were referred by the staff to be evaluated in the emergency department; and it took less of their time to be seen in the emergency department than it did to contact their primary care provider, only to then be told to go to the emergency department.

Fulde and Duffy[3] argued that patients who present frequently to emergency departments are a vulnerable and marginalised group perceived as not looking after themselves. Padgett et al [38] suggest that the emergency department is the "least appropriate setting for treating multiple co-morbidities". These authors also reported that people who feel powerless and isolated tend to be high emergency department users.

Hong *et al *[49] suggest that we need a radical paradigm shift to legitimise the role of emergency departments to include primary care services for those people on the lower end of the socioeconomic scale who tend not to have a primary care provider in the community. The counter opinion would be to restrict the role of emergency departments and to increase the availability of alternative primary care services and to thereby reduce emergency department overcrowding.

This research analysed emergency department data routinely collected by hospitals in the state of NSW, Australia, to identify patient related factors associated with three or more admissions a year and with admission for ACS conditions. The analysis was designed to answer questions about the impact on frequent readmissions of three sets of variables: demographic, clinical and arrival times and dates. The answers provide a basis for exploring the reasons underlying frequent readmissions. The research sets the scene for further examination of the reasons why people with chronic conditions are repeatedly admitted to hospital rather than seeking chronic disease management services.

## Methods

### Study design and site

In this study, we analysed 2008 data from the Emergency Department Information System collected at a regional hospital. The University of Wollongong/South Eastern Sydney Illawarra Health Service Medical Human Research Ethics Committee approved the research study (approval HE07/271). All patient data were de-identified. The study was carried out in a one hundred and fifty bed regional hospital in south eastern Australia which is part of a network with a major teaching and referral hospital. The hospital, funded by the State and Commonwealth Government, is located in a coastal regional urban area with a feeder population of around 100,000.

### Data Analysis

All statistical analyses were performed using SPSS statistical software (Version18; SPSS, Chicago, Illinois, USA). Two-sided P values of less than 0.05 were considered statistically significant.

### Predictor variables

The original variables were: patient identifier number, age, gender, date and time of arrival, country of birth, ICD 9 diagnosis, level of urgency based on the Australian Triage Classification [50], type of visit and mode of separation (treated in emergency or admitted). Type of visit is a local code referring to the type of emergency presentation: normal, planned return and unplanned return within 28 days of the original presentation. Each patient presentation was accorded one primary ICD 9 diagnosis based on the major reason for the presentation. Date and time of arrival variables were converted into hour, day and season of arrival.

The data was analysed either from the perspective of presentations in row-for-each-presentation format or from the perspective of patients in a row-for-each-patient format with nested data for each patient. The primary research question to uncover the patient characteristics associated with frequent readmissions required the row-for-each-patient format. However, preliminary univariate analysis was performed on the row-for-each-presentation format on the variables for which there were different or potentially different, values for each presentation: hour, day and season of arrival; urgency; unplanned return visit and diagnosis. Univariate analysis was also performed with the variables which were constant for each presentation (age, gender and country of birth).

### Primary outcome variable

The primary outcome variable was a binary variable of the number of admissions set with a cut off point at three or more admissions denoting frequent readmissions. Presenting patients judged to have conditions warranting admissions may be either admitted to the study hospital or to another hospital in the network if the bed supply is inadequate. All admissions, including those for which patients were placed in a bed in another hospital, were included in the sample. The number of admissions variable was obtained by separating the presentations for patients admitted, converting to the row-for-each-patient format and merging the admissions variable in to the main file.

### Regression models and manipulation of predictor variables

Two logistic regression models for multivariate analysis were constructed with the binary dependent variable. The independent variables were transformed by counting the number of occurrences in the nested variables for each patient and dividing by the total number of presentations for that patient resulting in a variable of the proportion of occurrences and used in the multivariate analysis. As the values in the proportion variables ranged from zero to one, they were treated as continuous covariates. The independent diagnosis variables included the proportion of the most common ICD 9 diagnosis codes for presentations resulting in frequent readmissions: neurosis, chronic heart failure, chronic obstructive pulmonary disease (COPD), dyspnoeas and chest pain.

In the second logistic regression model was created to determine the impact of diagnoses which are deemed suitable for non-hospital services. Ambulatory care sensitive conditions are defined as those which hospitalisation is considered potentially avoidable through preventive care and early disease management, usually delivered in an ambulatory setting, such as primary health care (for example by general practitioners or community health centres). For the second logistic regression model, the independent continuous proportion-of-diagnoses variables based on the ACS groupings: rapid onset conditions and chronic conditions [40]. were substituted for the continuous ICD 9 diagnoses proportion variables in the first model.

Logistic regression was considered the best option for analysis of the possible predictor variables because the dependent variable demonstrated Poisson distribution, with the variance higher than the mean, rendering linear regression models inappropriate [51,52]. However, there has been debate in the literature about the impact of intra-patient variability in emergency department presentations on the choice of statistical analysis. This has led to questioning of the appropriateness of logistic regression and the proposition that negative binomial regression should be used [53,54]. The results of the logistic regression were therefore checked against those of negative binomial regression. A backward elimination approach was adopted in the interest of achieving a parsimonious model [55,56]. The categorical variables, male gender and born in Australia both met the criterion of a minimum of ten cases in each category.

## Results

There were 21,956 presentations in 2008, of which 20% were admitted. Results of the univariate analysis on patient data in Table 1 indicate that there were significant differences between the general patient population and the frequent readmissions group in age, country of birth and gender. Table 2, presentation data, comparing the frequencies of the independent proportion variables in the general presenting population and presentations resulting in frequently readmissions, shows there were significant differences in all of the five most common diagnosis codes amongst frequently readmitted presentations and the "other" diagnoses, in urgent presentations, unplanned return visits and the chronic and "other" ambulatory care sensitive variables.

**Table 1:** Univariate analysis of patient characteristics comparing the general patient population with frequently readmitted patients 2008

	All patients (n = 15,806)	Patients admitted = >3 times (n = 144) 1% of all patients
**Males**	52%	48% p < 0.05^1^
**Mean age (SD, range)**	39 (26, 1-106)	66 (21, 2-96) p < 0.05
**Born outside Australia**	16%	27% p < 0.05^1^

^1^Chi square results

**Table 2:** Univariate analysis independent variables in the general presenting population compared to presentations resulting in frequent readmissions 2008.

**Variable group**	**Variable**	**General presenting population N = 21956**	**Presentations resulting in frequently readmissions N = 227**
**ICD9 diagnostic code**^**2**^	**Dyspnoeas (ICD 78609)**	2.3%	15.9% p < 0.01^3^
	**Neurotic disorder (ICD 3079)**	3.4%	10.6% p < 0.01
	**Chest pain (ICD 78650)**	4.4%	9.7% p < 0.01
	**Chronic obstructive pulmonary disease (ICD 496)**	0.7%	7% p < 0.01
	**Congestive heart failure (ICD 4280)**	0.3%	2.6% p < 0.01
	**All other ICD codes**	89.0%	54.2% p < 0.01
**Triage category**	**Urgent (Triage 1 & 2)**	5.9%	15.4% p < 0.01
**Visit type**	**Unplanned return visit**	4.3%	8.8% p < 0.01
**Season of arrival**	**Autumn**	24.9%	30.4% NS
	**Winter**	24.3%	28.6% NS
	**Spring**	25.0%	28.6% NS
	**Summer**	25.8%	12.3% p < 0.01
**Day of arrival**	**Weekend**	31.2%	28.2% NS
**Time of arrival**	**After hours**	49.0%	44.9% NS
**Ambulatory Care Sensitive Conditions**^**4**^	**ACS rapid onset**	3.9%	4.8% NS
	**ACS chronic**	1.3%	7.5% P < 0.01
	**ACS other**	94.8%	87.7% P < 0.01

^2^There were 1004 other ICD 9 codes with at least one presentation.

^3^Chi square results.

^4^ACS preventable was excluded because there was only one presentation in the general presenting population and none in the presentations resulting in frequent readmissions group.

Of the seasonal variables, only in summer was there a significant difference between the two groups. There were no differences observed in time or day of arrival.

Direct logistic regression uncovered the impact of the independent variables on frequent readmissions. Both models were initially set up with the independent variables gender, age, country of birth, and the proportion variables unplanned return visit, urgency, season, weekend, after hours and the diagnosis. Subsequently, stepwise backward elimination of the variable with the highest p value was performed to establish the most parsimonious model for the independent variables. The first model including the "proportion" variables for the five most common ICD 9 diagnoses was statistically significant with a *χ*^2 ^value of 278.3 (7 degrees of freedom, N = 15806, p < 0.01) indicating the model was able to distinguish between frequently readmitted and patients not frequently readmitted based on the definition of frequent admissions as three or more. As a whole, the ICD 9 diagnoses model explained up to 17.1% (R^2 ^Nagelkerk value) of the variance [57,58] in the dependent variable. As Table 3 reveals, seven of the independent variables, age, proportion of unplanned return visits, urgent, diagnoses of neurosis, chronic heart failure, COPD and dyspnoeas, made a statistically significant (p < 0.05) positive contribution to the model.

**Table 3:** Odds ratio and confidence limits for the variables having a significant impact on frequent readmissions in model one five most common ICD 9 diagnoses, results from logistic regression backwards elimination.

Variable	P value	Odds ratio	95% Confidence Interval for odds ratio	
			Lower	Lower
Age (years)	.000	1.046	1.036	1.055
Proportion of urgent (Triage categories 1 and 2)	.001	2.363	1.416	3.942
Proportion of neuroses (ICD 9 code 3079)	.000	13.430	7.435	24.258
Proportion of congestive heart failure (ICD 9 code 4280)	.027	4.720	1.189	18.744
Proportion of chronic obstructive pulmonary disease (ICD 9 code 496)	.000	8.807	3.857	20.107
Proportion of dyspnoeas (ICD 9 code 78609)	.000	6.095	3.425	10.845
Proportion of unplanned return visits within 28 days of previous visit	.000	14.381	5.487	37.693
Constant	.000	.000		

In the second model, a similar logistic regression analysis was performed substituting three variables of the proportion of preventable, rapid onset and chronic ACS conditions for the diagnosis variables in the first model. The other independent variables were similar to those used for model one. Overall, the ACS model was statistically significant with a *χ*^2 ^value of 192.8 (4 degrees of freedom, N = 15806, p < 0.01) indicating that the model could distinguish between frequently readmitted patients and non-frequently readmitted patients. As a whole, the ACS model explained up to 12.3% (Nagelkerk R^2 ^value [57]) of the variance of the dependent variable. In model two, four of the independent variables age, proportion of unplanned return visits, urgency and proportion of ACS chronic conditions made a statistically significant (p < 0.05) positive contribution to the model (see Table 4 The results of negative binomial regression models confirmed the logistic regression models.

**Table 4:** Odds ratio and confidence limits for the variables having a significant impact on frequent readmissions in model two ambulatory care sensitive conditions, results from logistic regression backwards elimination.

Variable	P value	Odds ratio	95% Confidence Interval for odds ratio	
			Lower	Upper
Age (years)	.000	1.043	1.035	1.052
Proportion urgent (Triage 1 & 2)	.001	2.320	1.407	3.825
Proportion unplanned return visits within 28 days of previous visit	.000	13.861	5.414	35.485
Proportion ACS Chronic conditions	.002	3.271	1.519	7.046
Constant	.000	.001		

## Discussion

The majority of presentations to the emergency department were by patients who had a single presentation in the year. The patients under investigation in this study were the one hundred and forty four frequently readmitted patients who had three or more admissions in a year. The univariate analysis showed significant differences in all the independent variables except the seasons of autumn, winter and spring and time and day of arrival. However, the multivariate analysis identified age, chronic conditions of neurosis, COPD, dyspnoeas and chronic heart failure, ACS chronic conditions, urgency and unplanned return visit as being associated with frequent readmissions. The study highlights the advantage of the multivariate analysis using the patient rather than presentation perspective to separate factors important in frequent readmissions.

Using the framework of Andersen's model of health service utilisation, the results can be considered in terms of patient demographics, clinical significance, access to services and individual patient preferences. The finding that age significantly influenced frequent readmissions is in line with other studies [31-33] These studies also reported that other demographic factors, sex and ethnicity, are associated with readmissions. However, there was no discernible sex difference, nor was there a difference between Australian born and those born outside Australia, in our study.

The type of condition is important in readmissions. Our model looked specifically at the most common conditions in presentations by patients admitted to a hospital bed. The diagnoses of neurosis, COPD, dyspnoeas and chronic heart failure, all serious conditions, were associated with frequent readmissions. Chest pain in our model was not associated with frequent readmissions. Although the regression models did not include a variable for severity of illness *per se*, other variables point to severity. The triage level specifically indicates urgency and more urgent conditions are generally more severe.

Unplanned return visits within 28 days of the previous visit for the same condition are reported by all public hospitals on a monthly basis to the NSW Department of Health. This indicator is considered to be important in assessing the quality of hospital care [59] on the basis that the underlying reason for the return visit may be related to premature discharge. Although the incidence of unplanned return visits is low, this is a predictor of frequent readmission and therefore worthy of further detailed investigation. An early warning system to flag unplanned return visits would assist in addressing their needs in a more timely way and reducing the burden on emergency services. Our findings in relation to unplanned return visits are in line with those reported for a US study on the Triage Risk Screening Tool [60].

The finding that frequent readmissions are associated with diagnoses of COPD, dyspnoeas and chronic heart failure is consistent with the notion that patients experiencing the symptoms associated with these conditions are more likely to believe their condition is serious enough for them to choose hospital care rather than primary care outside the hospital setting.

Seasonal variations in arrivals might have been expected because of the differences in temperature influencing the incidence of respiratory exacerbations. The fact that there were no seasonal variations is difficult to explain.

Patients with ACS chronic conditions were significantly more likely to be frequent readmissions. This finding could mean that access to primary care services for preventable conditions (immunisation and nutritional interventions) and rapid onset ACS conditions is adequate and access to chronic disease management services is not. Alternatively, the findings could indicate that patients are choosing hospital services over primary care services for chronic disease management.

Frequent readmissions for neurosis might be associated with reduced access to community based mental health services in the area. Another interpretation of this finding is that patients are voting with their feet expressing a preference for hospital services over community care.

Does poor access to other services contribute to frequent readmissions? Time and day of arrival had no significant impact on frequent readmissions. It might be expected that the more frequent emergency department users would opt for after hours or weekend visits when other community based services are less available. Our findings tend to negate any notion that access to after-hours and weekend services is an issue. However, the finding that ACS chronic conditions were associated with frequent readmissions suggests there are access issues at play. Although alternative general or specialist medical and chronic disease management services exists, there may be access difficulties other than the time of availability of services. Access factors such as, availability, accessibility, accommodation, affordability and acceptability, need to be further explored before we have a definitive answer on the impact on frequent readmissions. In particular, transport, location of community-based services and waiting times would act as barriers to access.

In our analysis, age and type of illness influenced readmissions. The impact of access issues on frequent readmissions is less clear. The other factors which could influence readmissions are related to patient preferences for the type of services, for example, general and specialist medical and chronic disease management services. If the uptake of these alternative services were to be increased, readmissions rates would be reduced. Further research to explore patient choice of services would be of benefit to the problem of frequent readmissions.

On the basis of the findings presented in this study, the question of whether readmissions are preventable was not directly answered, but the evidence from other studies shows that hospitalisation of chronic disease patients can be reduced by a range of targeted interventions to improve chronic disease management [45].

Logistic regression models were developed to identify the characteristics associated with frequent readmissions. The reliability of the predicting factors identified is determined by the robustness of the models. Although the overall fit of the model was significant, the Nagelkerk R^2 ^values of less than 20% were relatively low. However, other studies of patient utilisation and clinical data have quoted similarly low Nagelkerk R^2 ^values in logistic regression models [61,62].

This study is limited by the fact that it involves data pertaining to one hospital only. Although the results may not be generalisable to all hospitals, they are relevant to similar sized hospitals in non-metropolitan urban areas. Comparison with other hospitals with a similar role would lend weight to these findings. Another limitation is the purpose of the data collection. The data forms part of the Emergency Department Information System collected by all NSW hospitals. The accuracy of the recording of primary diagnosis in the data base is complicated by the fact that many of the older patients have multiple comorbidities and the reason for their presentation may not be apparent until test results are available. It was not possible to make any estimation of socioeconomic status from the data available. However, the area which the hospital services, although it contains some affluent pockets, is generally a low socioeconomic area [63]. As this study examines emergency department data only there is an inbuilt selection bias. We did not examine the use of other primary care services by people who did not access emergency department services. An expanded study to include the patterns of emergency department and community based primary care, particularly general practitioner services, is needed to develop population-based solutions to emergency department overcrowding. Another possible limitation is the failure of logistic regression analysis to take into account the intra-patient variability [51-54]. To address this issue we have compared the results of logistic and negative binomial regression models and found similar results. The relatively wide confidence intervals for some of the variables reduce the precision of the estimates. The study is also limited by the lack of a specific measurement of severity of illness.

## Conclusions

This study of routinely collected hospital data identified the factors associated with frequent readmissions with the aim of exploring the reasons behind readmissions. Strategies to identify and improve access to alternative non-emergency department services for neuroses, COPD, dyspnoea and chronic heart failure patients, including chronic disease self management and case coordination services, would reduce readmissions. An early warning system for the unplanned return visit patients would allow for special strategies to be put in place to address the needs of these patients. Possible community-based services access barriers such as transport, location of community-based services and waiting times should be explored and reduced. This study has opened up the possibility that a patient's preference for hospital over non-hospital services might influence readmissions and warrants further investigation.

## Competing interests

The authors declare that they have no competing interests.

## Authors' contributions

SEK, SMD, UWJ and MFH planned the research study. SMD and MFH supervised the PhD work. SEK carried out the initial analysis. All authors reviewed and approved the final manuscript.

## Pre-publication history

The pre-publication history for this paper can be accessed here:

http://www.biomedcentral.com/1472-6963/10/216/prepub
